# Decision-making for prenatal genetic screening: how will pregnant women navigate a growing number of aneuploidy and carrier screening options?

**DOI:** 10.1186/s12884-021-04282-7

**Published:** 2021-12-04

**Authors:** Ruth M. Farrell, Madelyn Pierce, Christina Collart, Meng Yao, Marissa Coleridge, Edward K. Chien, Susannah S. Rose, Mary Lintel, Uma Perni, Brownsyne Tucker Edmonds

**Affiliations:** 1 OB/GYN and Women’s Health Institute, Cleveland Clinic, 9500 Euclid Avenue, Cleveland, OH 44195 USA; 2 Genomic Medicine Institute, Cleveland Clinic, Cleveland, OH USA; 3 Center for Bioethics, Cleveland Clinic, Cleveland, OH USA; 4 Quantitative Health Science Department, Cleveland Clinic, Cleveland, OH USA; 5 Office of Patient Experience, Clinical Transformation, Cleveland Clinic, Cleveland, OH USA; 6 Department of OB/GYN, Indiana University, Indianapolis, IN USA

**Keywords:** Prenatal aneuploidy screening, Prenatal carrier screening, Decision-making, Patient education, Genomics

## Abstract

**Background:**

Prenatal genetic screens, including carrier screening (CS) and aneuploidy screening (AS), comprise an important component of reproductive healthcare delivery. Clinical practice guidelines emphasize the importance of informed decision-making and patient’s preferences regarding the use of these screens. Yet, it is unclear how to achieve this ideal as prenatal genetic screening options rapidly become more complex and increasingly available to patients. With increased complexity and availability of reproductive testing options, decision-support strategies are critical to prepare patients to consider AS and/or CS.

**Methods:**

A self-administered survey evaluated knowledge and decision-making preferences for expanded carrier (CS) and aneuploidy (AS) prenatal screening. The survey was administered to participants before their first prenatal visit to assess baseline decision-making needs and preference at the initiation of prenatal care. Analysis was approached as a descriptive process.

**Results:**

Participants had similar familiarity with the concepts associated with AS compared to CS; mean knowledge scores for CS was 0.59 [possible range 0.00 to 1.00] and 0.55 for AS. Participants reported preferences to learn about a range of conditions, including those with severe or mild impact, childhood-onset, and adult-onset. Decision-making preference with respect to learning about the associated disease phenotypes for the contained on AS and CS panel shifted with the complexity of the panel, with a greater preference to learn about conditions post-test compared pre-test education as panels increased from 5 to 100 conditions.

**Conclusion:**

Patients’ baseline knowledge of prenatal genetic screens coupled with evolving decision-making preferences presents challenges for the delivery of prenatal genetic screens. This calls for the development and implementation of innovative approaches to support pregnant patients’ decision-making commensurate with advances in prenatal genomics.

## Background

Prenatal genetic screening, comprising both aneuploidy screening (AS) and carrier screening (CS), is a core component of evidence-based obstetric healthcare delivery. However, it has become extremely difficult for pregnant women to navigate their prenatal genetic screening options in an informed fashion, for two reasons. First, advances in technology have greatly increased the number of screening options available to patients [1]. Second, clinical practice guideline changes have led to a large increase in the number of patients offered these screens during pregnancy, specifically universal screening approaches that have expanded upon previous age-based or ethnicity-based screening approaches [2–4]. Consequently, it is necessary now, more than ever, to develop effective approaches to ensure that patients can best make informed and preference-based decisions about their use.

One source of confusion for patients is that AS and CS provide different kinds of risk information about the impact of the identified variant on the pregnancy and future reproductive decision-making. AS focuses on identifying chromosomal aneuploidy (e.g., trisomy 21) and other genetic abnormalities such as microdeletions [5–9]. In contrast, CS focuses on identifying heritable genetic mutations that are present in the pregnant woman and can be passed to the offspring [2]. As conditions such as cystic fibrosis are heritable, the results of CS may impact decisions about the current pregnancy and any future reproductive decisions [10–14].

It is important to address this because previous studies have demonstrated that patients commonly struggle with understanding key informational aspects of both AS and CS and often lack the health literacy and numeracy skills to interpret and personalize the risk information generated by prenatal genetic screening [15–24]. In addition, a significant number of patients do not present for preconception care and in doing so, miss the opportunity to take advantage of genetic screening for reproductive planning prior to pregnancy [25–32].

Given these challenges, we conducted a study to evaluate both patients’ knowledge of prenatal genetic screening and their decision-making preferences for screening when offered as part of an expanded screening panel.

## Materials and methods

We conducted a cross-sectional study to examine pregnant patients’ knowledge and decision-making preferences for AS and CS when offered separately and in a combined single screening panel. The study was approved by the Cleveland Clinic Institutional Review Board. All components of the study experiments were performed in accordance with relevant human subject protection guidelines and regulations. Informed consent was obtained from all subjects. Study inclusion criteria were women, 18 years or older, who scheduled an initial obstetric appointment at one of the outpatient clinical practices of the Cleveland Clinic who were able to read and speak English and provide consent for research participation. Eligible participants were sent a recruitment letter describing the study procedures and a link to complete a self-administered survey via REDCap survey [Version 9.5.22] [33, 34].

Data were collected using a self-administered survey. The survey was administered to participants before their first prenatal visit to assess baseline decision-making needs and preferences at the initiation of prenatal care. The survey was administered via REDCap Survey which could be accessed on a computer or mobile device.

The survey was developed based on prior validated instruments and in conjunction with experts in prenatal genetics, obstetrics, and decision-making. The survey was composed of three sections. The first section focused on knowledge of CS and AS. These items were developed using clinical practice guidelines that outline the components of an informed decision-making process [35–39]. Knowledge questions were designed as a series of closed-ended items, with a true or false statement followed by response options of “Agree,” “Disagree”, and “I am not sure” for each knowledge item. The second section focused on decision-making preferences. One set of questions examined patient preferences for learning about conditions which different implications for quality of life for a child, using 5-point Likert scale responses. These questions did not define the quality of life but asked the participant to select the response that best aligned with her notions of mild, moderate, or severe. Another set of close-ended questions assessed patients’ preferences with respect to when they would want to learn about the conditions contained on an AS or CS panel. The third section collected participants’ demographic information and reproductive history information. The instrument was pilot tested with a representative sample of patients using a cognitive interview process and continued until no new points of revision were noted. The survey was revised based on those responses. A final version was developed for field use.

Analysis was approached as a descriptive process. A codebook was used to determine correct and incorrect answers to the knowledge questions. Overall knowledge scores were calculated based on the correctness of 24 knowledge questions for CS and 20 questions for AS. Possible overall scores range from 0.00 to 1.00. Other approximately normally distributed continuous measures were summarized using means and standard deviations and compared using t-tests. Continuous measures that showed a departure from normality and ordinal measures were summarized using medians and quartiles and compared using Wilcoxon rank-sum tests or the Kruskal-Wallis test. Categorical factors by age and reproductive history for knowledge items were summarized using frequencies and percentages and were compared using Pearson’s chi-square tests or Fisher’s Exact tests and educational levels using ANOVA. Pearson’s chi-square test and Kruskal-Wallis test were used to examine categorical factors for preference items. Analysis was conducted using SAS (version 9.4, The SAS Institute, Cary, NC). A *p* < 0.05 was considered statistically significant.

## Results

We approached 736 patients to participate in the study between May and November 2019. In total, 267 agreed to participate (36.3% participation rate). Of that, 75.2% of the surveys were completed. The mean age of participants used for analysis (201) was 32 ± 4 years old, and the majority had at least one prior pregnancy (67.2%), had a college degree or higher (75.1%), and classified themselves as Caucasian (87.1%) (Table 1).

**Table 1:** Demographics

Demographics of Participants	Total (***n*** = 201)
**Age**	31.9 ± 4.1
Non-AMA (< 35)	150 (74.6)
AMA (≥35)	51 (25.4)
**Race**	
White	175 (87.0%)
Black	13 (6.5%)
Asian	6 (3.0%)
American Indian or Alaska Native	1 (0.5%)
Other	6 (3.0%)
Hispanic or Latino (Yes)	10 (5.0%)
**Education Level**	
Some High School	1 (0.5%)
High School Graduate or GED	12 (6.0%)
Associates Degree, technical degree, or some college	37 (18.4%)
College graduate	75 (37.3%)
Graduate or professional degree	76 (37.8%))
**Marital Status**	
Single	14 (7.0%)
Currently Married	169 (84.1%)
Committed relationship	18 (9.0%)
**Faith (Yes)**	129 (64.2%)
Christian	119 (92.2%)
Muslim	3 (2.3%)
Hindu	1 (0.78%)
Other	6 (4.7%)
**Prior Pregnancies**	
No prior pregnancy	66 (32.8%)
One or more Prior pregnancy	135 (67.2%)

### Knowledge of concepts associated with aneuploidy and carrier screening

The mean overall score for knowledge items (e.g., knowledge of the indications for screening, knowledge of post-screen options) was 0.58 [0.42, 0.75] for CS knowledge, and for AS knowledge was 0.60 [0.40, 0.75] (Table 2). Participants were more familiar with the concepts associated with AS compared to CS. With respect to conditions, higher knowledge levels were noted for autosomal aneuploidies, with 62.2% of patients correctly identifying AS identified the risk of trisomy 21, 48.8 and 42.3% correctly identified the inclusion of trisomy 18, and trisomy 13. There was less familiarity with some of the newer screening applications: only 24.5% identified that AS could be performed for Turner syndrome and 22.0% for Klinefelter syndrome. Overall, lower knowledge levels were noted for conditions associated with CS compared to AS: 43.8% were familiar with cystic fibrosis, 39.3% with sickle cell anemia, 33.8% with spinal muscular atrophy, and 26.4% with thalassemia.

**Table 2:** Knowledge items for carrier screening and aneuploidy screening

Baseline knowledge of Carrier Screening and Aneuploidy Screening		
**Knowledge of:**	**Carrier**	**Aneuploidy**
**Overall score median [Q1, Q3]**	0.58 [0.46, 0.75]	0.55 [0.40, 0.75]
**Overall score mean ± sd**	0.59 ± 0.18	0.55 ± 0.23
	**% (N) correctly answered**	**% (N) correctly answered**
**Conditions recommended for screening**	Cystic fibrosis 43.8% (88)	Trisomy 21,125 (62.2%)
	SMA 33.8% (68)	Trisomy 18,177 (48.8%)
	Thalassemia 26.4% (5)	Trisomy 13 85 (42.3%)
	Sickle cell 39.3% (79)	Turner 53 (26.4%)
		Klinefelter 44 (21.9%)
**Etiology of recessive condition or aneuploidy condition**	30.8% (62)	81.6% (164)
**Recommended action for a positive screen result**	53.2% (107)	57.7% (116)
**Implications for future pregnancies**	34.8% (70)	80.6% (162)
**Recommendation for universal offering of screen**	46.3% (93)	45.3% (91)
**Etiology of the genetic condition**	51.2% (103)	69.2% (139)
**Reproductive History risk for condition**	85.1% (171)	82.6% (166)
**Maternal age as a risk factor for a condition**	(No increase with age) 23.9% (48)	(Increase with age) 79.1% (159)
**Inheritance (heritable or sporadic condition)**	34.8% (70)	27.4% (55)

Baseline knowledge of the genetic factors associated with fetal chromosomal aneuploidy and recessive conditions was low (Table 2). With respect to understanding the etiology of aneuploidy or a recessive condition, 30.8% of the participants correctly responded to a question about the percent chance of having a child with a recessive condition if both biological parents were carriers, and 81.6% correctly responded to a question about the potential for a fetus to have a chromosomal condition even if there is no family history of the condition. When asked about post-screen testing options following a positive screen result, 57.7% correctly identified options to follow a positive aneuploidy screen, and 53.2% correctly identified options to follow a positive CS result. With respect to the impact of one of these genetic conditions for a future pregnancy, most participants (80.6%) recognized that aneuploidy is commonly spontaneous and would not affect future pregnancies (independent of maternal age). In contrast, only 34.8% understood that the identification of an autosomal recessive variant could affect a future pregnancy. Participants had little familiarity with current recommendations that all patients should be offered CS (46.3%) and AS (45.3%). Just over half of the participants recognized what kind of genetic variant would result in a recessive condition (51.2%) or aneuploidy (69.2%). The majority correctly identified that there was still a risk of the fetus being diagnosed with a recessive condition (85.1%) or aneuploidy (82.6%) if the biological parents did not have a prior pregnancy affected by these conditions. Most thought that maternal age was a risk factor for both CS and AS. Few patients could identify if conditions screened for were inherited (CS) or sporadic (AS). The level of education proved a significant factor for predicting knowledge of CS or AS, with higher education levels associated with higher knowledge levels for CS (*p* < 0.001) and AS (*p* = 0.066). No associations were noted based on age, parity, or ethnicity.

### Decision-making preferences for aneuploidy and carrier screening

With respect to decision-making preferences, several trends for AS and CS were observed. As panels increased in the number of tested conditions, participants noted a greater preference to learn about genetic conditions only after screen results were available. When AS panels screened for 5 conditions, 32.8% noted preferences for pre-test access to information about all of the conditions on the panel, and 19.9% wanted to learn only about conditions they were at risk for, while 47.2% noted a preference to defer learning to the post-test period. Preferences shifted as panels contained more conditions. When panels increased to contain 50 conditions, fewer participants (17.9%) noted preferences to learn about all of the conditions on the panel, while the number of participants who preferred post-test education increased (62.1%). Of note, participants who preferred to limit pre-test education to conditions at which they were at risk for did not change significantly as panels increased from 5 conditions to 100 conditions. Similar trends were noted for preferences regarding information exchange and the number of conditions contained on a CS panel (Table 3).

**Table 3:** Preferences of when to receive education in regards to various conditions based on number of screened conditions

Number of conditions being screened for				
	5	10	50	100
**Aneuploidy Screening Panels** % (N) Aneuploidy screening knowledge correct %				
Preference for pre-test information: All conditions on a panel	32.8% (66) 0.60	29.3% (59) 0.60	17.9% (36) 0.50	18.4% (37) 0.50
Preference for pre-test information: Only conditions at-risk for	19.9% (40) 0.55	19.9% (40) 0.60	19.9% (40) 0.55	16.9% (34) 0.55
Preference for post- test information	47.2% (95) 0.55	50.7% (102) .50	62.1% (125) .62	64.6% (130) .60
**Carrier Screening Panels** % (N) Carrier screening knowledge correct %				
Preference for pre-test information: All conditions on a panel	28.8% (58) .58	27.8% (56) .58	15.0% (30) .58	14.5% (29) .50
Preference for pre-test information: Only conditions at-risk for	20.8% (42) .58	20.8% (42) .56	20.5% (41) .63	20.0% (40) .56
Preference for post- test information	50.2% (101) .63	51.2% (103) .63	64.5% (129) .64	65.5%(131) .63

With respect to the type of condition that could be identified, the time of disease onset was important. Most reported their preferences as very or extremely important to learn about a condition that would result in a child’s death soon after birth (88.0%) and in the first year of life (88.0%). A majority also wanted to learn about childhood-onset genetic conditions with mild impact on child’s quality of life (QoL) (69.0%), moderate impact on child’s QoL (78.9%), severe impact on child’s QoL (89.0%), and conditions for which the QoL unknown (73.4%). More than half also noted a preference for prenatal genetic screens that would convey information about an adult-onset condition (60.5%) (Table 4).

**Table 4:** Importance of Information about Different Conditions: Very Important or Extremely Important responses

Condition Type	N (%) (***N*** = 201)
**Childhood onset - Death soon after birth**	176 (88.0%)
**Childhood onset - Death first year of life**	178 (89.0%)
**Childhood onset - Mild impact on child’s QoL**	138 (69.0%)
**Childhood onset - Moderate impact on child’s QoL**	157 (78.9%)
**Childhood onset – Severe impact on child’s QoL**	178 (89.0%)
**Childhood onset - Impact on QoL unknown**	146 (73.4%)
**Adult onset condition**	121 (60.5%)

Education levels were associated with these preferences: women with high-school/some college/technical degree reported that it was very or extremely important to learn about conditions that would mildly affect QoL (*p* = 0.008) and conditions that would fully affect QoL would not be known until after birth (*p* = 0.025) compared to women with college or a graduate degree. Prior pregnancy experience was associated with different preferences. Participants without previous pregnancy reported greater preferences to learn about a condition that would lead to the death of a child soon after birth (97.0% vs. 83.6%; *p* = 0.007) or severely affect the QoL of a child (97.0% vs. 85.1%; *p* = 0.012), compared to participants who had a prior pregnancy (Table 5).

**Table 5:** Importance of Information about Different Conditions

Factor	Total N	Some high school to some college (***N*** = 50)	College graduate (***N*** = 75)	Graduate or professional degree (***N*** = 76)	***p***-value
**I would want to learn about a medical condition that would result in the death of a child soon after birth.**	200				0.19^a^
Not at all important		0 (0.00)	0 (0.00)	1 (1.3)	
Slightly/Somewhat		4 (8.0)	7 (9.3)	12 (16.0)	
Very/Extremely important		46 (92.0)	68 (90.7)	62 (82.7)	
**I would want to learn about a medical condition that would result in the death of a child in the first year of childhood.**	200				0.40^a^
Not at all important		0 (0.00)	0 (0.00)	1 (1.3)	
Slightly/Somewhat		5 (10.0)	6 (8.0)	10 (13.3)	
Very/Extremely important		45 (90.0)	69 (92.0)	64 (85.3)	
**I would want to learn about a a medical condition that would mildly affect the quality of life of a child.**	200				***0.008***^***a***^
Not at all important		1 (2.0) ^3^	3 (4.0)	3 (4.0) ^1^	
Slightly/Somewhat		7 (14.0)	19 (25.3)	29 (38.7)	
Very/Extremely important		42 (84.0)	53 (70.7)	43 (57.3)	
**I would want to learn about a a medical condition that would moderately affect the quality of life of a child.**	199				0.091^a^
Not at all important		1 (2.0)	1 (1.3)	2 (2.7)	
Slightly/Somewhat		4 (8.2)	16 (21.3)	18 (24.0)	
Very/Extremely important		44 (89.8)	58 (77.3)	55 (73.3)	
**I would want to learn about a medical condition that would severely affect the quality of life of a child.**	200				0.31^a^
Not at all important		0 (0.00)	0 (0.00)	1 (1.3)	
Slightly/Somewhat		3 (6.0)	8 (10.7)	10 (13.3)	
Very/Extremely important		47 (94.0)	67 (89.3)	64 (85.3)	
**I would want to learn about a medical condition that’s full effects on the quality of life of the child will not be known until after birth.**	199				***0.025***^***a***^
Not at all important		1 (2.0) ^3^	2 (2.7)	2 (2.7) ^1^	
Slightly/Somewhat		5 (10.2)	19 (25.3)	24 (32.0)	
Very/Extremely important		43 (87.8)	54 (72.0)	49 (65.3)	
**I would want to learn about a medical condition that may develop when a child grows up and is an adult.**	200				0.071^a^
Not at all important		1 (2.0)	4 (5.3)	5 (6.7)	
Slightly/Somewhat		13 (26.0)	25 (33.3)	31 (41.3)	
Very/Extremely important		36 (72.0)	46 (61.3)	39 (52.0)	

Statistics presented as Mean ± SD, N (column %)

^a^ *p* values determined by the Kruskal-Wallis test

^1^ Significantly different from Some high school to some college

^2^ Significantly different from Graduate or professional degree

Post-hoc pairwise comparisons were done using Bonferroni adjustment

## Discussion

This study highlights the challenges that prenatal genetic screening presents for both pregnant patients and their obstetric healthcare providers. Our findings raise important clinical and ethical questions about how best to ensure that patients can make autonomous, informed decisions about their screening options. That includes not only ensuring that patients have the information and resources they need to make decisions about those options, but also understanding patients’ needs and values in the process so that the final decision best reflects their goals.

While knowledge levels for both kinds of screening were low at baseline, participants had greater familiarity with AS at the onset of prenatal care compared to CS. Knowledge deficits were noted across several informational categories, including the conditions contained in these screenings, the risk factors for a fetus being diagnosed with chromosomal or genomic variant, the interpretation of screen results, and the implications of those risk assessments on their pregnancy and future reproductive decision-making. In many regards, these findings were expected. Prior studies have demonstrated patient knowledge deficits and challenges for informed decision-making for prenatal AS factors have persisted despite the continued growth of cfDNA screening [40–43]. Other studies have demonstrated deficits in patient knowledge and decision-making for CS [18, 36, 44–47]. While some of these are due to patient-related factors, others are due to healthcare-related factors, including impediments in provider knowledge and adherence to evidence-based guidelines about their use [48–55].

Our findings indicate that it will require significant time and effort for healthcare providers to overcome the patients’ deficits in knowledge. This is problematic, given that providers have increasingly less time during clinical encounters to engage in lengthy conversations. These results point to the increasing workload required at the first prenatal visit to ensure patients are prepared to make informed prenatal genetic screens decisions. Ideally, prenatal genetic screening options should be offered at the onset of prenatal care. While final decisions about the use of prenatal genetic screens may not be made at the initiation of prenatal care, the counseling that occurs at this time sets the stage for patients’ informed access to genetic information about the pregnancy. In reality, these discussions do not occur in isolation, but during the time at which other important aspects of prenatal healthcare delivery must be discussed (e.g., the role of folic acid, immunization), with the addition of significant topical issues (e.g., COVID-19) [1]. These factors affect how much time and effort can be allocated during this visit to support patients’ understanding of screening and diagnostic testing options and the implications of their choices on the outcome of the pregnancy [56, 57].

In addition, as prenatal genetic panels become increasingly complex, it will become more difficult to structure and individualize a patient-centered decision-making process. For instance, we found that participants preferred to learn about all of the conditions on a panel when there were only 5–10 conditions; however, when the number of conditions increased to 50 or 100, participants preferred to defer learning about conditions until the post-test period. While some authors have suggested using a generic informed consent process for expanded panels [58], this approach may not meet patients’ needs for informed and autonomous decision-making for panels with less than 50 conditions, as it is necessary to ensure patients understand how screening accuracy may decrease as the number of conditions on a panel increases [59]. In addition, participants in this study found personal value in panel tests that can provide information about a range of different identifiable variants that can affect the quality of life or viability. This preference included not only information about variants associated with severe childhood-onset conditions but also those associated with adult-onset conditions and conditions which have an uncertain impact on the quality of life, although screening is not recommended for such conditions [ 3, 14, 18]. Thus, there may be a need to align patient preferences with evidence-based guidance, an effort complicated by the continued availability of expanded panels that depart from current guidelines.

We recognize that issues related to patients’ health literacy and knowledge with respect to prenatal genetic screening have been an ongoing clinical challenge. This situation calls for integrating innovative approaches, resources, and technological solutions as we reevaluate how to best support patients’ informed decision-making about an expanding array of prenatal genetic screening options at the onset of prenatal care [60]. For instance, patient engagement software is a new approach used in other areas of healthcare driven by patient preferences and needs [61]. These types of programs can present information with decision aids and then, using the input of patients, respond and adapt to patient preferences and needs to provide more personalized education. Such an approach may reduce the challenges to condensing these processes in a single visit. In addition, using programming pathways, information delivery can be automated so that it can be delivered at different time points in prenatal care delivery. This would be an optimal approach to initiate decision-making once a pregnant patient schedules her initial prenatal visit and then at set times over the prenatal episode in conjunction with screening and diagnostic testing milestones. It would also help prepare patients to discuss their options with a genetic counselor by introducing them to genetic screening concepts in preparation for that visit. Further research is needed to determine how to integrate innovative approaches into prenatal care delivery to meet the challenges posed by genomics. This should include studies that seek to understand patients’ goals and preferences, not only with prenatal genetic screening but also with the process that leads to the final decision to use or decline this option, a factor that will be critical with advances in screening technology.

As study data were collected using a self-administered survey among eligible patients who elected to participate, it is crucial to consider the impact of response and selection bias among the types of patients who completed the survey, particularly given our low response rate. However, when we tested for statistical differences between responders and non-responders, we did not find significant differences between groups. In conducting this study, we sought a broad demographic representation in our recruitment efforts; yet, most respondents were > 35 years of age, self-described Caucasian, with higher education levels, and from the same geographic areas. Knowledge and decision-making preferences of women of different ages, education levels, or race/ethnic groups remain uncertain. Thus, further research is needed to elucidate these important issues. Despite these limitations, our study sheds light on significant challenges facing patients, healthcare providers, and healthcare systems with the clinical implementation of new prenatal genomic screens. These findings, in conjunction with studies demonstrating the role of healthcare technology in navigating healthcare decisions, may highlight the role of an innovative way to approach the persistent issues related to patients’ health literacy and knowledge. There is a growing awareness of the role of patient education and decision-making tools that allow for patients’ asynchronous learning before and after the clinical visit in which prenatal genetic screens and diagnostic tests are discussed. There is also the increasing role of artificial-intelligence based tools that can provide a more “human” and natural conversational language experience of patients who use these technologies. These may be important new avenues to support patients as they consider an increasing number of prenatal genetic screening options. While such tools may not take the place of discussions with a healthcare provider or genetic counselor, they may be adjunct to synergizing resources in the goal to personalize care to patients. At the same time, there is also the opportunity to revisit the formative discussions that patients may have with their healthcare provider. This also calls for exploring the role of individual and small group counseling to determine what approach may best align with the needs of patients as they consider their prenatal genetic screening options.

## Conclusion

In conclusion, we have identified significant clinical and ethical challenges to ensuring that patients have the information and decision support needed to make informed choices about their prenatal genetic screening options during pregnancy. Thus, it is necessary for healthcare providers and systems to revisit how to structure the medical decision-making processes during the onset of prenatal care in a way that is commensurate with advances in prenatal genomics. It is unlikely that one approach will solve the challenges presented by emerging genomic applications in prenatal care. Instead, we must look for a combination of resources to best prepare for the individualized needs of patients as they consider their prenatal genetic screening and diagnostic testing options. This presents a prime opportunity for innovation, not just in leveraging advances in digital educational tools and resources, but approaches to optimize the important discussions that patients may have with their healthcare provider over the course of the pregnancy.

## Data Availability

The datasets generated and/or analyzed during the current study are not publicly available due to the original design and validation of the project but are available from the corresponding author on reasonable request.

## References

[1] Kilpatrick SJ, Papil L-A, Macones GA, editors. Chapter 6, Antepartum Care. In: Guidelines for Perinatal Care [Internet]. 8th ed. Elk Grove Village: American Academy of Pediatrics; 2017. p. 150-226. [cited 2020 July 8]. Available from: https://reader.aappublications.org/guidelines-for-perinatal-care-8th-edition/168.

[2] American College of Obstetrics and Gynecologists (2017). Committee opinion No. 691: carrier screening for genetic conditions. Obstet Gynecol.

[3] Gregg AR, Skotko BG, Benkendorf JL (2016). Noninvasive prenatal screening for fetal aneuploidy, 2016 update: a position statement of the American College of Medical Genetics and Genomics. Genet Med..

[4] American Society for Reproductive Medicine & American College of Obstetricians and Gynecologists (2019). Prepregnancy counseling: committee opinion number 762. Fertil Steril.

[5] American College of Obstetrics and Gynecologists (2016). Practice Bulletin No. 163: screening for fetal aneuploidy. Obstet Gynecol.

[6] del Gil M, Nicolaides K (2019). Implementation of maternal blood cell free DNA testing in early screening for aneuploidies. J Matern Fetal Med.

[7] Mennuti MT, Chandrasekaran S, Khalek N, Dugoff L (2015). Cell-free DNA screening and sex chromosome aneuploidies. Prenat Diagn.

[8] Wapner RJ, Babiarz JE, Levy B (2015). Expanding the scope of noninvasive prenatal testing: detection of fetal microdeletion syndromes. Am J Obstet Gynecol.

[9] Vora NL, OʼBrien BM. (2014). Noninvasive prenatal testing for microdeletion syndromes and expanded Trisomies: proceed with caution. Obstet Gynecol.

[10] Neitzel D, Glass S, Leahey J, Faulkner N (2019). Carrier screening: should evaluating more genes be the standard of care?. Fertil Steril.

[11] Grody WW, Cutting GR, Klinger KW (2001). Laboratory standards and guidelines for population-based cystic fibrosis carrier screening. Genet Med..

[12] American College of Obstetrics and Gynecologists (2017). Committee opinion no. 690 summary: carrier screening in the age of genomic medicine. Obstet Gynecol.

[13] Norton ME (2017). Expanded carrier screening: a rational approach to screening for rare diseases. Obstet Gynecol.

[14] Edwards JG, Feldman G, Goldberg J (2015). Expanded carrier screening in reproductive medicine—points to consider. Obstet Gynecol.

[15] Piechan JL, Hines KA, Koller DL (2016). NIPT and informed consent: an assessment of patient understanding of a negative NIPT result. J Genet Couns.

[16] Sheinis M, Bensomon K, Selk A (2017). Patients’ knowledge of prenatal screening for trisomy 21. J Genet Couns.

[17] Seven M, Akyüz A, Eroglu K (2016). Women’s knowledge and use of prenatal screening tests. J Clin Nurs.

[18] Kraft SA, Duenas D, Wilfond BS, Goddard KAB (2019). The evolving landscape of expanded carrier screening: challenges and opportunities. Genet Med..

[19] Poels M, Koster M, Boeije H (2016). Why do women not use preconception care? A systematic review on barriers and facilitators. Obstet Gynecol Surv.

[20] Smith SK, Cai A, Wong M (2018). Improving women's knowledge about prenatal screening in the era of non-invasive prenatal testing for Down syndrome - development and acceptability of a low literacy decision aid. BMC Pregnancy Childbirth..

[21] Gates EA (2004). Communicating risk in prenatal genetic testing. J Midwifery Womens Health.

[22] Shieh C, Halstead JA (2009). Understanding the impact of health literacy on women’s health. J Obstst Gynecol Neonatal Nurs.

[23] Dormandy E, Tsui EY, Marteau TM (2007). Development of a measure of informed choice suitable for use in low literacy populations. Patient Educ Couns.

[24] Lee S, Holden D, Webb R, Ayers S (2019). Pregnancy related risk perception in pregnant women, midwives & doctors: a cross-sectional survey. BMC Pregnancy Childbirth.

[25] M’hamdi HI, van Voorst SF, Pinxten W (2017). Barriers in the uptake and delivery of preconception care: exploring the views of care providers. Matern Child Health J.

[26] American Academy of Family Physicians (2015). Preconception Care (Position Paper).

[27] Bello JK, Goutham R, Stulberg DB (2015). Trends in contraceptive and preconception care in United States ambulatory practices. Fam Med.

[28] Ohio Department of Health (2010). Preconception health and women in Ohio - Online Brochure.

[29] Phillippi JC (2010). Women's perceptions of access to prenatal care in the United States: a literature review. J Midwifery Womens Health.

[30] MacDorman MF, Declercq E, Thoma ME (2017). Trends in maternal mortality by sociodemographic characteristics and cause of death in 27 states and the District of Columbia. Obstet Gynecol.

[31] Creanga AA, Bateman BT, Kuklina EV, Callaghan WM (2014). Racial and ethnic disparities in severe maternal morbidity: a multistate analysis, 2008-2010. Am J Obstet Gynecol.

[32] Grody WW, Thompson BH, Gregg AR (2013). ACMG position statement on prenatal/preconception expanded carrier screening. Genet Med.

[33] Harris PA, Taylor R, Thielke R (2009). Research electronic data capture (REDCap) – a metadata-driven methodology and workflow process for providing translational research informatics support. J Biomed Inform.

[34] Harris PA, Taylor R, Minor BL (2019). REDCap consortium, the REDCap consortium: building an international community of software partners. J Biomed Inform.

[35] Ready K, Haque IS, Srinivasan BS, Marshall JR (2012). Knowledge and attitudes regarding expanded genetic carrier screening among women’s healthcare providers. Fertil Steril.

[36] Ong R, Howting D, Rea A (2018). Measuring the impact of genetic knowledge on intentions and attitudes of the community towards expanded preconception carrier screening. J Med Genet.

[37] Lazarin GA, Haque IS (2016). Expanded carrier screening: a review of early implementation and literature. Semin Perinatol.

[38] Ormond KE, Banuvar S, Daly A (2009). Information preferences of high literacy pregnant women regarding informed consent models for genetic carrier screening. Patient Educ Couns.

[39] American College of Obstetricians and Gynecologists’ Committee on Practice Bulletins—Obstetrics; Committee on Genetics; Society for Maternal–Fetal Medicine (2016). Practice Bulletin No. 162: prenatal diagnostic testing for genetic disorders. Obstet Gynecol.

[40] Kuppermann M, Norton ME, Thao K (2016). Preferences regarding contemporary prenatal genetic tests among women desiring testing: implications for optimal testing strategies. Prenat Diagn.

[41] Kaimal AJ, NortonME KM (2015). Prenatal testing in the genomic age: clinical outcomes, quality of life, and costs. Obstet Gynecol.

[42] Portocarrero ME, Giguère AM, Lépine J (2017). Use of a patient decision aid for prenatal screening for Down syndrome: what do pregnant women say?. BMC Pregnancy Childbirth..

[43] Kuppermann M, Pena S, Bishop JT (2014). Effect of enhanced information, values clarification, and removal of financial barriers on use of prenatal genetic testing: a randomized clinical trial. JAMA..

[44] Shea TL (2020). Informed decision making regarding prenatal aneuploidy screening. J Obstet Gynecol Neonatal Nurs.

[45] Fakih A, Spector-Bagdady K (2019). Should clinicians leave ‘expanded’ carrier screening decisions to patients?. AMA J Ethics.

[46] Darcy D, Tian L, Taylor J, Schrijver I (2011). Cystic fibrosis carrier screening in obstetric clinical practice: knowledge, practices, and barriers, a decade after publication of screening guidelines. Genet Test Mol Biomarkers.

[47] Rothwell E, Johnson E, Mathiesen A (2016). Experiences among women with positive prenatal expanded carrier screening results. J Genet Couns.

[48] Brewer J, Demers L, Musci T (2017). Survey of US obstetrician opinions regarding NIPT use in general practice: implementation and barriers. J Matern Fetal Neonatal Med.

[49] Agatisa PK, Mercer MB, Mitchum A (2018). Patient-centered obstetric Care in the age of cell-free fetal DNA prenatal screening. J Patient Exp.

[50] Macri CJ, Gaba ND, Sitzer LM (2005). Implementation and evaluation of a genetics curriculum to improve obstetrician-gynecologist residents' knowledge and skills in genetic diagnosis and counseling. Am J Obstet Gynecol.

[51] Driscoll DA, Morgan MA, Schulkin J (2009). Screening for Down syndrome: changing practice of obstetricians. Am J Obstet Gynecol.

[52] Chan WV, Johnson JA, Wilson RD, Metcalfe A (2018). Obstetrical provider knowledge and attitudes towards cell-free DNA screening: results of a cross-sectional national survey. BMC Pregnancy Childbirth..

[53] Suskin E, Hercher L, Aaron KE, Bajaj K (2016). The integration of noninvasive prenatal screening into the existing prenatal paradigm: a survey of current genetic counseling practice. J Genet Couns.

[54] Farrell RM, Agatisa PK, Mercer MB, Mitchum AG, Coleridge MB (2016). The use of noninvasive prenatal testing in obstetric care: educational resources, practice patterns, and barriers reported by a national sample of clinicians. Prenat Diagn.

[55] Dondorp W, De Wert G, Bombard Y (2015). Non-invasive prenatal testing for aneuploidy and beyond: challenges of responsible innovation in prenatal screening. Eur J Hum Genet.

[56] Johnston J, Farrell RM, Parens E (2018). Supporting women’s autonomy in prenatal testing. Obstet Gynecol Surv..

[57] Farrell RM, Agatisa PK, Michie MM (2019). The personal utility of cfDNA screening: pregnant patient experiences with cfDNA screening and views on expanded cfDNA panels. J Genet Couns.

[58] de Jong A, Dondrop WJ, de Diesmulders CEM (2010). Non-invasive prenatal testing: ethical issues explored. Eur J Hum Genet.

[59] Best S, Wou K, Vora N (2018). Promises, pitfalls and practicalities of prenatal whole exome sequencing. Prenat Diagn.

[60] Wapner RJ, Driscoll DA, Simpson JL (2012). Integration of microarray technology into prenatal diagnosis: counselling issues generated during the NICHD clinical trial. Prenat Diagn.

[61] Rosner BI, Gottlieb M, Anderson WN (2018). Effectiveness of an automated digital remote guidance and telemonitoring platform on costs, readmissions, and complications after hip and knee aathroplasties. J Arthroplast.

